# Aberrant expression of miR-214 is associated with obesity-induced insulin resistance as a biomarker and therapeutic

**DOI:** 10.1186/s13000-019-0914-1

**Published:** 2020-02-24

**Authors:** Fangxiao Cheng, Geheng Yuan, Jiao He, Yimin Shao, Junqing Zhang, Xiaohui Guo

**Affiliations:** 1 Department of Endocrinology, Peking University First Hospital, No. 8 Xishiku Street, Xicheng District, Beijing, 100034 China; 2 Department of Endocrinology, Baoding First Central Hispital, Baoding, 071000 Hebei Province China

**Keywords:** Obesity, Insulin resistance, MicroRNA-214, DDP4, Adipocyte

## Abstract

**Background:**

Insulin resistance (IR) in obesity is associated with the occurrence of metabolic and cardiovascular diseases. Dipepidyl peptidase 4 (DPP4) plays a pivotal role during the development of IR, and was found to be a target gene of microRNA-214 (miR-214) in our study. This study sought to assess the expression and clinical value of miR-214 in obese patients with IR, and investigate its therapeutic potential in obese rats and adipocytes with IR.

**Methods:**

Serum expression of miR-214 in obese patients with or without IR was estimated by quantitative real-time-PCR. A receiver operating characteristic curve was plotted to evaluate the diagnostic value of miR-214 in the patients. Obesity-induced IR animal and cell models were constructed, and the therapeutic ability of miR-214 was explored.

**Results:**

Serum expression of miR-214 was decreased in obese patients compared with the healthy controls, and the lowest expression was observed in the cases with IR. Downregulation of miR-214 was significantly correlated with the serum DPP4 levels and HOMA-IR of the patients upon IR conditions, and was demonstrated to perform diagnostic accuracy for distinguishing obese patients with IR from those without IR. In obesity-associated IR animal and cell models, the downregulation of miR-214 was also been detected. According to the measurement of glucose and insulin tolerance and glucose uptake abilities, we found that the overexpression of miR-214 could be used to alleviate IR in the IR models, especially when collaboratively used with DPP4 inhibitor vildagliptin.

**Conclusion:**

All data revealed that miR-214, as a regulator of DPP4, is decreased in obese patients with IR and may serve as a diagnostic biomarker. The upregulation of miR-214 could improve IR in obese rats and adipocytes, indicating that miR-214 has the therapeutic potential for obesity and IR.

## Introduction

Obesity becomes a major global health burden due to the comorbidities resulted from excessive adiposity, such as hyperglycemia, diabetes mellitus (DM) and insulin resistance (IR) [1]. The current lifestyle trends that characterized by caloric abundance and reduced physical activity leads to increased incidence of obesity-associated comorbidities [2]. IR is a frequently pathological condition occurred in obese individuals that the response of cells to the hormone insulin is weakened [3]. Chronic inflammation in obese patients is a critical cause of obesity-associated systemic IR [4, 5]. The presence of IR represents the dysfunction of glucose uptake and metabolism, leading to various metabolic and cardiovascular related diseases [6]. Thus, it is important to identify IR cases among obese patients and improve the therapeutic strategies for this pathological condition. Dipepidyl peptidase 4 (DPP4) is a serine protease and could increase of blood glucose by degradation of incretin hormones, leading to the occurrence of IR [7–9]. Vildagliptin belongs to a kind of incretin-based drug, which is an inhibitor of DPP4 and has been widely used for DM and IR. However, the patients with IR still have enormous demand for more efficient therapeutic methods.

MicroRNAs (miRNAs) are a group of small non-coding RNAs with regulatory roles of gene expression at the post-transcriptional level [10]. They are involved in the pathogenesis of various human diseases, including obesity, by regulating the expression of key genes of the disease progression [4, 11]. Thus, emerging evidence have focused on the function of miRNAs and implied that the regulation of miRNAs could be novel therapeutic strategies for the treatment of various diseases [12]. Currently, some members of miRNAs with aberrant expression patterns have been identified in adipose tissues, such as miR-148a [13], miR-150 [14], miR-377 [4] and miR-34a [5], which are associated with the obesity-induced inflammation and IR. In the present study, we demonstrated that miR-214 was a regulator of DPP4 in adipocytes. A previous study by Zhu et al. [15] has reported that miR-214 could mediate the promoting effects of lncRNA MEG3 in hepatic IR. Another study by Nigro et al. [16] gives evidence that miR-214 might be involved in the IR development in mouse aortic endothelial cells. However, the role of miR-214 in obesity-associated IR remains unclear. It is noticeable that the regulatory function of miR-214 in inflammatory response has been demonstrated, and it is determined as one of the key molecules that serve as potential therapeutic targets in inflammation-associated diseases [17]. Subsequent studies have confirmed the close relationship of miR-214 with inflammatory response in some diseases [18, 19]. Considering the pivotal role of DPP4 and inflammation in the development of IR, we deduced that miR-214 might also be associated with obesity-induced IR.

In this study, we aimed to investigate the expression profile of miR-214 in obese patients with or without IR, and evaluate the diagnostic value of miR-214 for the differentiation between IR cases from simple obese patients. In addition, we sought to construct obesity-related IR animal and cell models to further explore the therapeutic potential of miR-214.

## Materials and methods

### Patients and sample collection

The protocols of this study were approved by the Ethics Committee of Peking University First Hospital University. This study enrolled 120 obese patients, including 60 cases with IR and 60 cases without IR (simple obesity), from the hospital between 2016 and 2017. In addition, 40 healthy individuals were recruited, who underwent routine health examination in the hospital. None of the healthy controls had IR or any other metabolic diseases. All the participants provided the written informed consents. Venous blood samples were collected from the patients and healthy controls, and serum specimens were extracted from the blood using centrifugation. The fasting blood glucose (FBG) and fasting serum insulin (FINS) were measured and used for the calculation of homeostasis model of assessment for IR index (HOMA-IR).

### Cell culture and transfection

Mouse 3 T3-L1 pre-adipocytes were purchased from the American Type Culture Collection (ATCC, Manassas, USA) and cultured in Dulbecco’s modified Eagle’s medium (DMEM; Gibco, CA, USA) supplemented with 10% fetal bovine serum (FBS). For the differentiation of pre-adipocytes, the cells were incubated in induction medium I (10 μg/mL of insulin, 0.5 mM 1-methyl-3-isobutyl-xanthine and 1 μg/mL of dexamethasone in DMEM supplemented with 10% FBS) for 24 h after they spread to all the bottom of the culture plates. After a further 24 h incubation in fresh DMEM supplemented with 10% FBS, the cells were cultured with the induction medium II (10 μg/mL of insulin in DMEM with 10% FBS0 for 48 h. Lastly, the cells were cultured in fresh DMEM with 10% FBS, which was renewed 2 days once until the mature adipocytes accounting for 95% of the cells.

To regulate the expression of miR-214 in 3 T3-L1 cells, miR-214 mimic (ACAGCAGGCACAGACAGGCAGU), miR-214 inhibitor (ACUGCCUGUCUGUGCCUGCUGU) or miRNA negative control (miR-NC, UUCUCCGAACGUGUCACGU) (GenePharma, Shanghai, China) was transfected into the cells using Lipofectamine 2000 (Invitrogen, Carlsbad, CA, USA). The cells transfected with only transfection reagent were set as the mock group.

### RNA extraction and quantitative real-time-PCR (qRT-PCR)

Total RNA was extracted by Trizol reagent (Invitrogen, Carlsbad, CA, USA) according to the manufacturer’s instruction. Single stranded cDNA was synthesized from the RNA using a PrimeScript RT reagent kit (Takara, Shiga, Japan). The expression of miR-214 was measured using qPCR, which was carried out using a SYBR green I Master Mix kit (Invitrogen, Carlsbad, CA, USA) and the ABI Prism 7300 system (Thermo Fisher Scientific, Waltham, MA, USA). Following are the sequences of primer: miR-214 F: 5′-GCCGAGACAGCAGGCACAG-3′, R: 5′-CTCAACTGGTGTCGTGGA-3′; U6 F: 5′-CTCGCTTCGGCAGCACA-3′, R: 5′-AACGCTTCACGAATTTGCGT-3′. The relative expression values were calculated by 2^−ΔΔCt^ method and normalized to U6.

### Luciferase reporter assay

DPP4 was predicted as a target gene of miR-214 using TargetScan (http://www.targetscan.org/vert_72/). The 3′-UTR of DPP4 was combined into the psiCHCK-2 vector as the wild-type vector (DPP4-WT), and the mutant 3′-UTR was used to construct the mutant-type vector (DPP4-MT). The 3 T3-L1 cells were seeded into 24-well plates and co-transfected with miR-214 mimic, miR-214 inhibitor or miR-NC and DPP4-WT or DPP4-MT. Luciferase activity was estimated using a dual-luciferase reporter assay system (Promega, Madison, WI, USA).

### Enzyme-linked immune sorbent assay (ELISA)

The concentration of DPP4 was estimated using the Human sCD26 Platinum ELISA kit (eBioscience, Vienna, Austria) as per the manufacturer’s protocols.

### Obesity-induced IR animal model and treatment

Total of 36 Sprague-Dawley (SD) rats (males, 6 weeks) were purchased from Beijing Vital River Laboratory Animal Technology Company (Beijing, China). After a one-week adaptation, the rats were used for model construction. The protocols for animal experiments were approval by the Animal Ethics Committee of Peking University. The rats were randomly divided into 2 groups, including normal control (NC) group (*n* = 6) and IR model group (*n* = 30). The animals in the NC group were fed with regular diet (10% fat). The rats in the IR model group were fed with D12492 high-fat diet (HFD; 60% fat) for obesity-associated IR model construction. The body weight (BW) of the rats was recorded during the 12 weeks of feeding, as well as the FBG and FINS.

The IR model group was further divided into 4 groups: IR control group (*n* = 6), vildagliptin (VG) group (n = 6), miR-214 mimic group (n = 6) and VG + miR-214 mimic group (n = 6). The animals in the IR control group were given normal saline by gavage. The rats in the VG group were oral injected with 3 mg/kg VG (Novartis, Basel, Switzerland). In the miR-214 mimic group, Lentivirus (100 μL, 2 × 10^7^ TU/mL) with miR-214 mimic were intraperitoneally injected in the rats. In the VG + miR-214 mimic group, the animal received both VG and miR-214 mimic treatments. The blood and omental adipose tissues were collected from the rats and stored at − 80 °C for further examination.

### Obesity-associated IR cell model and treatment

After differentiation, the mature 3 T3-L1 adipocytes were grouped into untreated group and palmitic acid (PA) group. The cells in untreated group received no treatment as controls. The cells in PA group received stimulation of 1 mM PA for the construction of IR cell model.

To evaluate the effects of miR-214 on IR development in vitro, the IR model cells were also divided into IR control (IR control-C) group, VG (VG-C) group, miR-214 mimic (miR-214 mimic-C) and VG + miR-214 mimic (VG + miR-214 mimic-C) group. In the VG-C group, IR model cells were treated with 50 nM DPP4 for 24 h. In the miR-214 mimic-C group, the miR-214 mimic was transfected into the adipocytes using Lipofectamine 2000 before 48 h of PA treatment. The cells in the VG + miR-214 mimic-C group were received the treatment with both VG and miR-214 mimic.

### Oral glucose tolerance test (OGTT) and intraperitoneal insulin tolerance test (IPITT)

OGTT and IPITT were performed to confirm the construction of IR animal model and to evaluate the effects of miR-214 on the development of IR. For OGTT, the animals free accessed to water and were fasted for 12 h, and then treated with 40% of glucose (2 g/kg BW) via oral injection. A glucose meter (ACCU-CHEK Performa Roche, Germany) was used to examine the blood glucose at the tip of the tail at 0, 30, 60, 90, 120 min after the administration of glucose. The area under the curve (AUC) was calculated to evaluate the sensitivity of islet β-cells. For IPITT, the rats free accessed to water and were fasted for 12 h, and then treated with insulin (0.75 IU/kg BW) through intraperitoneal injection. A glucose meter was used to examine the blood glucose at the tip of the tail at 0, 30, 60, 90, 120 min after the administration of glucose. The AUC value was also computed to evaluate the insulin sensitivity.

### Glucose uptake assay

To analyze the IR conditions of the adipocytes, glucose uptake assay was performed using a glucose colorimetric/fluorometric assay kit (Biovision, Inc., Milpitas, CA, USA). In briefly, the cells were incubated with or without insulin (100 nM) at 37 °C for 20 min. Then the glucose assay buffer was added into the cells and incubated at 37 °C for 10 min. The absorbance at 570 nm was measured by a microplate reader (BioTek, Beijing, China).

### Statistical analysis

All the data in this study were expressed as mean ± SD and analyzed using SPSS 21.0 software (SPSS Inc., Chicago, IL) and GraphPad Prism 7.0 software (GraphPad Software, Inc., USA). Comparisons between groups were assessed by Student’s t test or one-way ANOVA followed by Turkey’s post hoc test. Pearson correlation analysis was used to analyze the correlation between two parameters. The diagnostic potential of miR-214 was evaluated using a receiver operating characteristic curve (ROC). A difference with a *P* < 0.05 was considered statistically significant.

## Results

### DPP4 is a direct target gene of miR-214

**Fig. 1:**
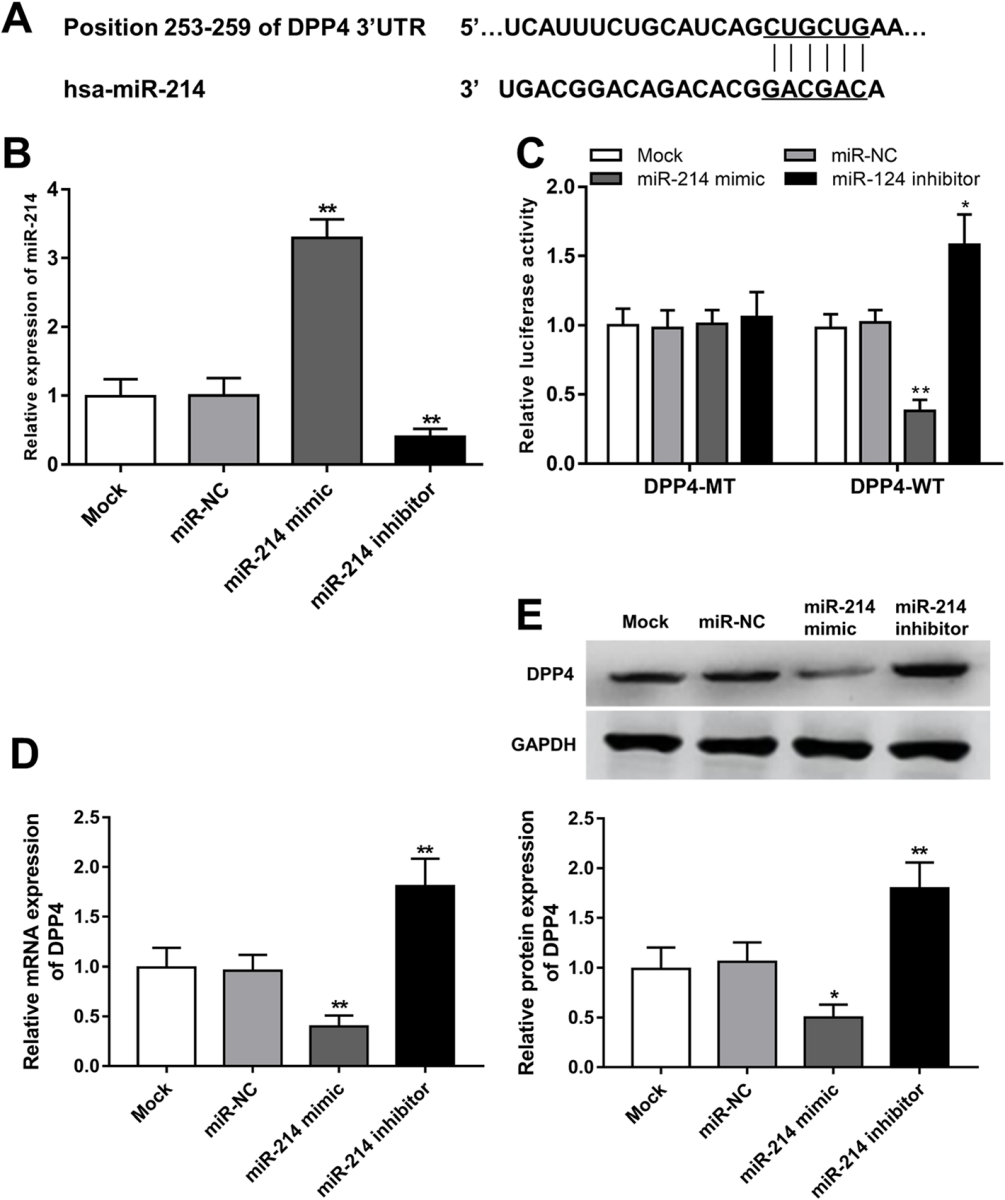
miR-214 served a regulator of DPP4 in adipocytes. **a.** The 3′-UTR of DPP4 had complementary sequence of miR-214. **b.** Expression of miR-214 in adipocytes was upregulated by miR-214 mimic, but was downregulated by miR-214 inhibitor. **c.** Relative luciferase activity of the wild-type 3′-UTR of DPP4 was significantly suppressed by the overexpression of miR-214, but was promoted by the knockdown of miR-214. **d** and **e**. Effect of miR-214 on the mRNA **d** and protein **e** expression of DPP4. *P < 0.05 and **P < 0.01

According to the bioinformatics analysis, we realized that DPP4 is a target gene of miR-214 with a complementary sequence of miR-214 in its 3′-UTR (Fig. 1a). To prove the interaction between miR-214 and DPP4, a luciferase activity assay was carried out. The expression of miR-214 in the adipocytes was regulated by miR-214 mimic and miR-214 inhibitor, which separately led to the upregulation and downregulation of miR-214 (all *P* < 0.01, Fig. 1b). The luciferase activity results shown in Fig. 1c indicated that the luciferase activity of DPP4-WT group was suppressed by the overexpression of miR-214, but was enhanced by the knockdown of miR-214 (all *P* < 0.05). However, no significant difference in luciferase activity was observed in the DPP4-MT group (all *P* > 0.05). Additionally, the mRNA and protein expression changes of DPP4 in adipocytes under dysregulation of miR-214 were further assessed. As shown in Fig. 1d and e, the expression of DPP4 in both mRNA and protein levels were suppressed by the overexpression of miR-214, but was promote by the knockdown of miR-214 (all *P* < 0.05).

### Serum expression of miR-214 in obese patients

**Fig. 2:**
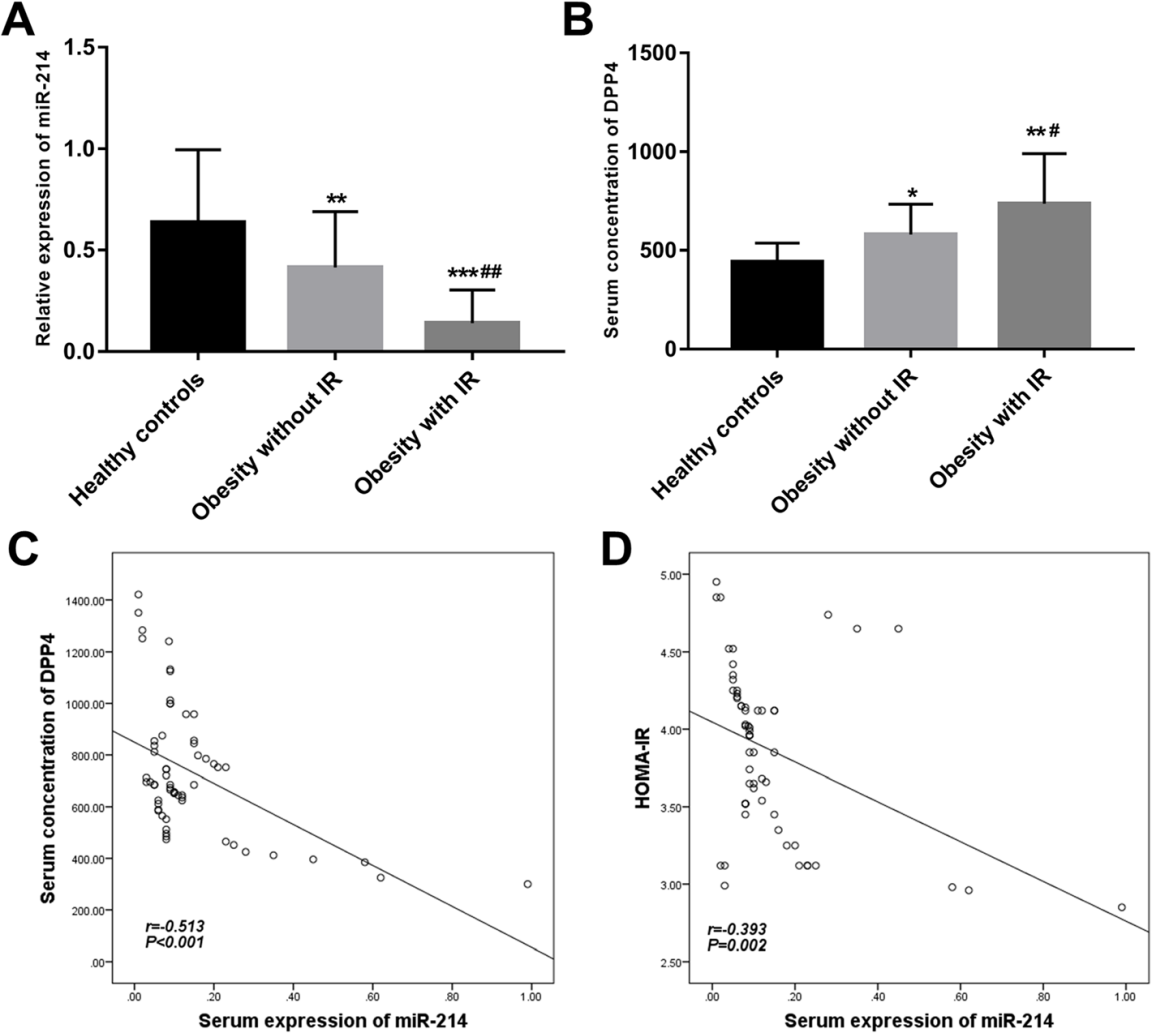
Serum expression of miR-214 in obese patients and its correlation with DPP4 levels and HOMA-IR. **a**. Serum expression of miR-214 was decreased in the obese patients compared with the healthy controls, and the lowest levels were observed in the obese cases under IR conditions. **b**. Serum concentration of DPP4 was increased in the obese patients, especially in the IR cases, compared with the healthy controls. **c**. A negative correlation between miR-214 expression and the concentration of DPP4 was found (r = − 0.513, *P* < 0.001). **d**. The serum expression of miR-214 was negatively correlated with the HOMA-IR of the obese patients with IR (r = − 0.393, *P* = 0.002)

Since miR-214 was proved to be a regulator of DPP4 in adipocytes, this study focused on the role of miR-214 in obese patients. According the qRT-PCR, serum expression of miR-214 was found to be significantly downregulated in the obese patients compared with the healthy controls, and the lowest expression levels of miR-214 were observed in the obese cases upon IR conditions (all *P* < 0.01, Fig. 2a).

### Correlation of miR-214 expression with DPP4 expression and HOMA-IR

As we all known that the expression of DPP4 exhibits an obviously increase in obese patients with IR, and HOMA-IR is one of the key indexes to reflect the conditions of IR. Thus, the correlation between the two indicators and the expression of miR-214 was analyzed. As shown in Fig. 2b, the serum concentration of DPP4 in IR positive obese patients was dramatically upregulated compared with the simple obese patients and healthy controls (all *P* < 0.05), and its level was negatively correlated with the expression of miR-214 (r = − 0.513, *P* < 0.001, Fig. 2c). As expected, a positive correlation was observed between the expression of miR-214 and HOMA-IR (3.86 ± 0.54) of the obese patients under IR conditions (r = − 0.393, *P* = 0.002, Fig. 2d).

### Diagnostic value of miR-214 in the obese patients

**Fig. 3:**
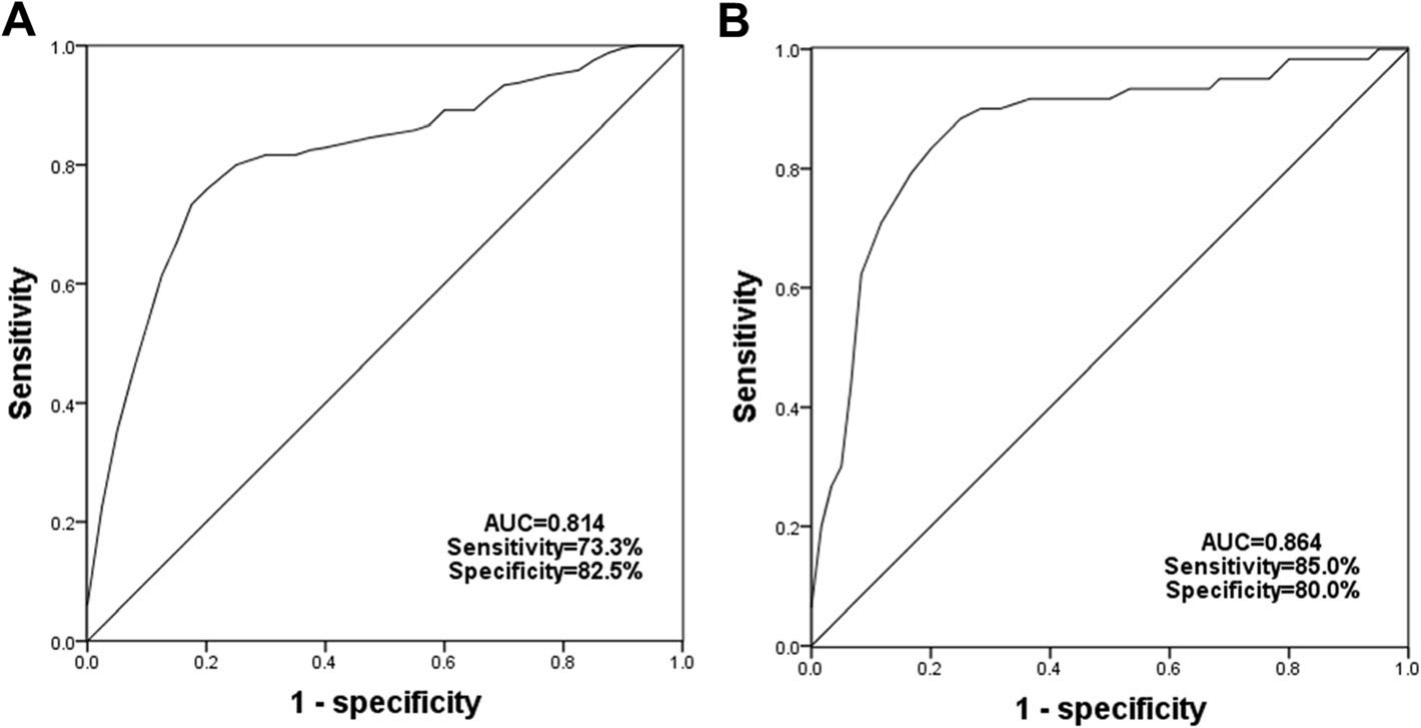
ROC curves plotted based on the expression of miR-214 in the obese patients. **a**. Serum expression of miR-214 could be used for distinguishing obese cases from healthy individuals with an AUC of 0.814. **b**. Serum expression of miR-214 had relative high diagnostic accuracy for the differentiation between obesity with IR and obesity without IR (AUC = 0.864, sensitivity = 85.0%, specificity = 80.0%)

Given the aberrant expression of miR-214 in the serum of the obese patients, we further investigated the clinical significance of the dysregulation. By the ROC analysis, we found that the decreased expression of miR-214 could be used to distinguish obese patients from the healthy individuals with the area under the curve (AUC) of 0.814 (cutoff value = 0.355, sensitivity = 73.3%, specificity = 82.5%) (Fig. 3a). Moreover, the diagnostic accuracy of miR-214 was also relative high for the differentiation between IR positive obese patients and the non-IR obese patients, and the AUC was 0.864, the sensitivity and specificity were respectively 85.0 and 80.0% at the cutoff value of 0.220 (Fig. 3b). Thus, we believed that the aberrant expression of miR-214 could be used for the diagnosis of IR cases from the obese patients.

### Establishment of obese animal model and adipocyte model with IR

**Fig. 4:**
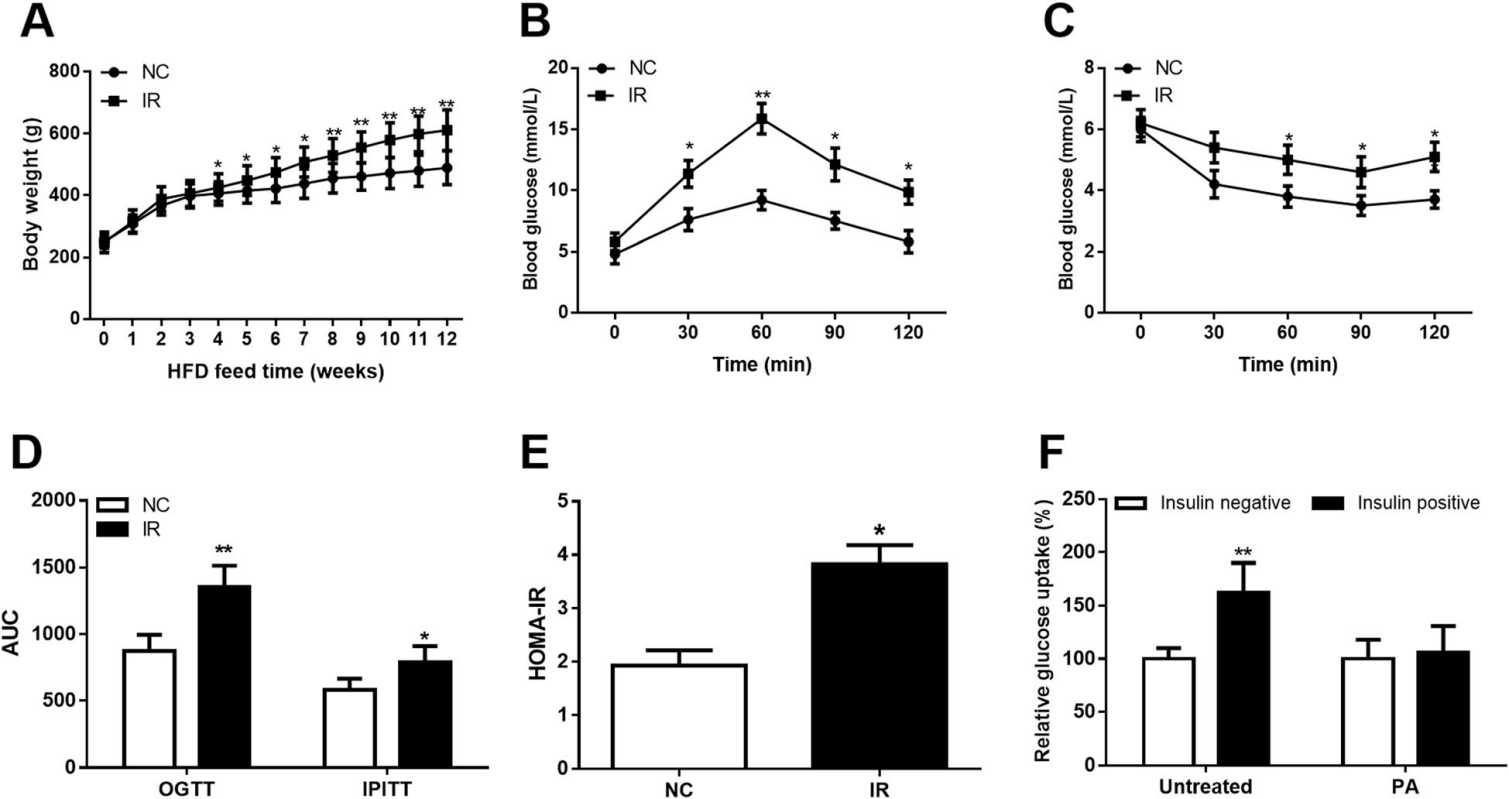
Establishment of obesity-associated IR animal and cell models. **a.** Body weight (BW) in IR animals was increased compared with the normal controls. **b.** Glucose tolerance was significantly impaired in the IR group compared with the NC group. **c.** Insulin tolerance in IR group was lower than that in the NC group. **d.** AUCs of IR group for OGTT and IPITT were all increased compared with that in NC group. **e.** IR animal had higher HOMA-IR than that in the normal rats. **f.** Insulin-stimulated glucose uptake was suppressed by PA treatment in the 3 T3-L1 cells compared with the normal adipocyte. *P < 0.05 and **P < 0.01

After 12 weeks of HFD, the IR animal model was constructed. The BW of the rats was elevated in the IR group compared with the NC group (*P* < 0.05, Fig. 4a). The glucose and insulin tolerances were both impaired in the IR animals when compared to the normal controls, which evidenced by the results of OGTT and IPITT results and the subsequently computed AUCs (all *P* < 0.05, Figs. 4b **- 4D**). By using the FBG and FINS, the HOMA-IR was calculated and shown a remarkably increase in the IR rats compared with the NC (*P* < 0.05, Fig. 4e). The data above suggested that the obesity-induced IR was successfully constructed.

To evaluate the availability of the IR cell model, the glucose uptake assay was conducted. As shown in Fig. 4f, the insulin-stimulated glucose uptake ability of the adipocytes was obviously suppressed by the treatment of PA (*P* < 0.01), suggesting that we obtained the IR cell model.

### Expression of DPP4 and miR-214 in the IR animal and cell models

**Fig. 5:**
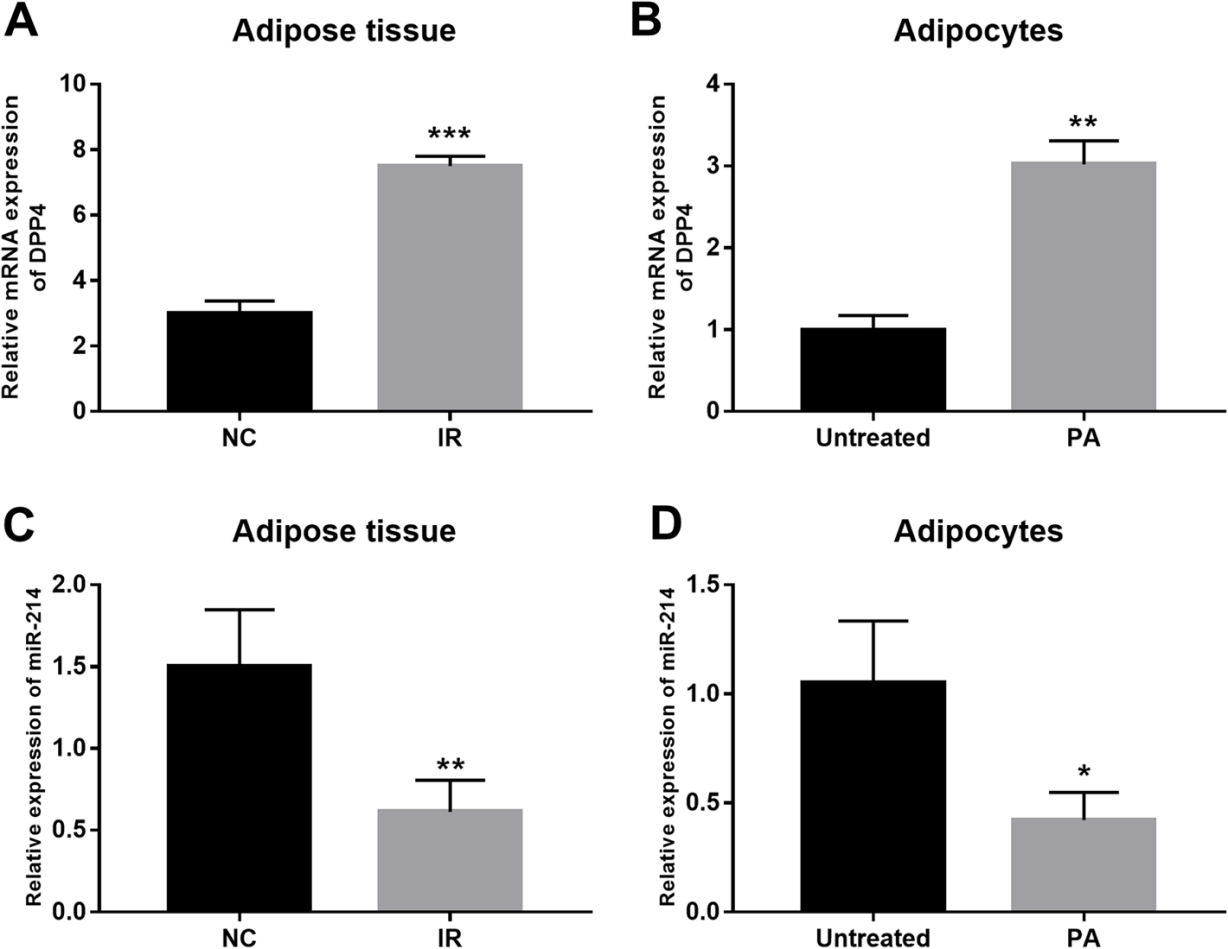
Expression of DPP4 and miR-214 in the IR animal and cell models. **a**. The expression of DPP4 was increased in adipose tissues of IR rats compared with the normal controls. **b**. The adipocytes treated with PA had a higher DPP4 expression than those untreated adipocytes. **c**. Decreased expression of miR-214 was observed in the adipose tissues of the IR rats compared with that in the normal rats. **d**. Expression of miR-214 was reduced in the adipocytes after the treatment of PA. *P < 0.05 and **P < 0.01, ***P < 0.001

Following the successful model construction, the expression of DPP4 and miR-214 was examined in the IR animals and cells. The qRT-PCR data revealed that the mRNA expression of DPP4 was expectably increased in both adipose tissue and adipocytes under IR conditions (all P < 0.01, Fig. 5a and b). The expression levels of miR-214 in the adipose tissues of the IR animals and the IR adipocytes were markedly declined compared with the corresponding normal controls (all *P* < 0.05, Fig. 5c and d), which is consistent with the expression results in the clinical samples.

### Overexpression of miR-214 and DPP4 inhibition improve IR conditions

**Fig. 6:**
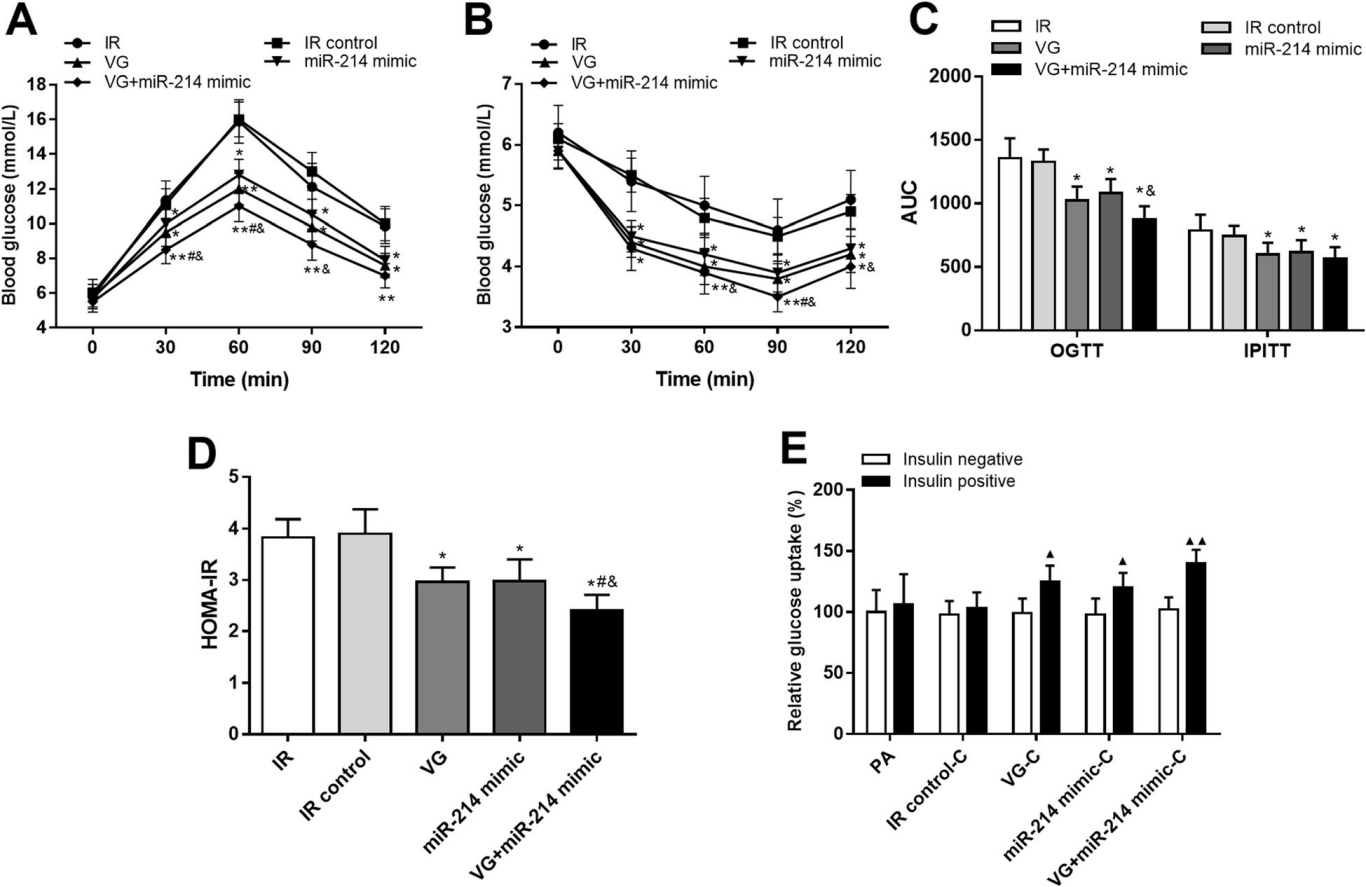
Effects of miR-214 on the IR conditions in the established IR models. **a-c.** VG treatment or the overexpression of miR-214 in IR rats rescued the glucose and insulin tolerance, and the combination of these two methods resulted more improved glucose and insulin tolerance. **d.** HOMA-IR of the IR animals was decreased by VG treatment or the upregulation of miR-214. **e.** Blocked insulin-stimulated glucose uptake by PA treatment was rescued by the treatment with VG or the overexpression of miR-214, and the combined therapy of VG treatment and miR-214 overexpression could further improve the glucose uptake ability. *P < 0.05 and **P < 0.01 compared to the IR group; ^#^*P* < 0.05 compared to the VG group; ^&^*P* < 0.05 compared to the miR-214 mimic group; ^▲^*P* < 0.05, ^▲▲^*P* < 0.01 compared to the insulin negative group

It is well known that VG is a widely used DPP4 inhibitor to improve IR. According to the OGTT and IPITT, we found that the impaired glucose and insulin tolerance were rescued by VG treatment in IR rats (*P* < 0.05). Similarly, we also observed the improved glucose and insulin tolerance by overexpression of miR-214 (*P* < 0.05). Moreover, the glucose and insulin tolerance were further improved by combined therapy with VG treatment and miR-214 overexpression (*P* < 0.05, Figs. 6-5a-c). In addition, the HOMA-IR was decreased by VG treatment or miR-214 overexpression as compared with the IR animals, and the lowest HOMA-IR was observed in the animals received combined treatment with VG and miR-214 mimic (*P* < 0.05, Fig. 6d).

In IR cell model, we examined the glucose uptake to evaluate the effects of miR-214 on IR conditions. As shown in Fig. 6e, the blocked insulin-stimulated glucose uptake by PA treatment was rescued following the treatment with VG (*P* < 0.05). The treatment results of miR-214 overexpression was consistent with the VG, which also showed increased glucose uptake ability after the treatment (*P* < 0.05). More importantly, we observed more improved glucose uptake ability in the cells received combined therapy with VG treatment and miR-214 overexpression (*P* < 0.01).

## Discussion

Obesity is considered one of the most common metabolic diseases, and leads to various comorbidities [20]. IR, as one of the obesity-associated pathological condition, is involved in the occurrence of metabolic and cardiovascular disorder [21]. Thus, there is an urgent need for the effective strategies for IR treatment in the obese patients. DPP4 is a key molecular during the occurrence and development of IR in T2DM patient [22]. Thus, some therapeutic methods focused on DPP4 have been improved, such as the established DPP4 inhibitor VG [23]. Therefore, we believed that the exploration of potential molecules that related with the regulation of DPP4 might contribute to the development of IR treatment.

According to the bioinformatics prediction, this study found that DPP4 was one of the target gene of miR-214. Moreover, miR-214 was further proved to be a regulator of DPP4 in the adipocytes. Accumulated evidence demonstrate that miRNAs could exist pivotal regulatory effects in the pathogenesis of various diseases mediated by the target genes [24–27]. In the disease progression of IR, some members of miRNAs with critical roles have also been reported [28]. For example, a study by Ke et al. [29] indicated that miR-721 was downregulated during the treatment of IR by astragalus polysaccharides. Dou and his colleagues shown that miR-338-3p expression was associated with the development of IR by targeting PP4R1 and regulation of PP4 [30]. The deficiency of miR-150 in non-alcoholic fatty liver disease resulted in decreased IR through regulation of CASP8 [31]. The expression of miR-543 was found to be elevated in TNF-α induced IR cells, and its reduction could alleviate IR by targeting SIRT1 [32]. All these aforementioned studies indicated that the functional miRNAs could be used as therapeutic targets for the treatment of IR.

miR-214 has previously reported to be deregulated in hepatic IR [15] and muscle cell IR [33], and it is determined as a key molecule in the regulation of inflammation [17–19], but rare evidence for its role in obesity-induced IR. In this study, the expression of miR-214 in obese patients with IR was estimated and its clinical value in diagnosis was further evaluated. Compared with the healthy controls, we found that the serum expression of miR-214 was decreased in the obese patients, and that the lowest expression was detected in the obese cases upon IR conditions. Additionally, serum miR-214 expression was demonstrated to be negatively correlated with serum DPP4 concentration and the HOMA-IR, indicating that the aberrant expression of miR-214 might be involved in the development of obesity-associated IR. Furthermore, the ROC curves based on the serum expression of miR-214 was plotted and revealed that the decreased miR-214 expression could be used as a diagnostic biomarker for screening obese patients from healthy individuals, and had a relative high diagnostic accuracy for the differentiation between the obese patients with IR and the obese cases without IR. Taken together, the data of the clinical research indicated that the downregulated expression of miR-214 serves a candidate diagnostic biomarker for the obese patients under IR conditions.

Since the direct regulatory effect of miR-214 on DPP4 was proved in our study, a hypothesis was conducted that miR-214 might play a key role in the disease progression of obesity-associated IR. Thus, the functional role of miR-214 was investigated in the IR animal and cell models. After the successful model construction, a significant decrease in the expression of miR-214 was also observed in the models, which was consistent with the clinical results. By the in vivo regulation with miR-214 mimic transduction, the impaired glucose and insulin tolerance in the obese rats was obviously ameliorated, and the increased HOMA-IR was also improved by the overexpression of miR-214. In the IR cell model, the inhibited glucose uptake ability induced by PA treatment was markedly abrogated by the upregulation of miR-214. Interestingly, we found that the beneficial effects of miR-214 were similar to the treatment of VG, which is a widely used inhibitor of DPP4 in clinical practices [34]. More importantly, the combination treatment with miR-214 overexpression and VG resulted in a more significant improvement compared with the two alone in the IR animal and cell models, suggesting that the therapeutic efficacy of VG was promoted by the upregulation of miR-214. Therefore, we believed that the upregulation of miR-214 could be used to improve the obesity-induced IR, and the combined use of miR-214 and VG might be a more efficient therapeutic method for the treatment of obese patients under IR conditions.

Although the present study provided new insight on the expression profile, clinical significance and functional role of miR-214 in obesity-associated IR, the molecular mechanisms underlying the role of miR-214 remain elusive. Our study demonstrated that DPP4 was a direct target gene of miR-214, indicating that miR-214 might exist its beneficial effects by downregulating the expression of DPP4. It is well known that increased DPP4 contributes to the cleavage of incretin, leading to the inhibition of insulin secretion [35]. In addition, Ghorpade et al. have reported that hepatocyte-secreted DPP4 promoted the development of IR in obesity by the activation of the inflammation in the macrophage through targeting CAV1 [36]. However, whether the mechanisms mentioned above also mediated the function of miR-214/DPP4, or if the miR-214 existed the protective effects via other genes or signaling, are still unclear and warrant further investigations.

## Conclusion

In conclusion, this study demonstrated the significantly decreased expression of miR-214 in the serum of obese patients with IR, and the downregulation of miR-214 served an efficient diagnostic biomarker for the screening of IR cases from the obese patients. Furthermore, we also provided evidence for the beneficial effects of miR-214 in the IR obese animals and IR adipocytes, indicating that miR-214 may be a potential therapeutic target and combined therapy with miR-214 and VG may be a novel therapeutic method for the treatment of obesity and IR.

## Data Availability

All data generated or analysed during this study are included in this published article.

## Abbreviations

ATCC: American Type Culture Collection
AUC: Area under the curve
AUC: Area under the curve
BW: Body weight
DM: Diabetes mellitus
DMEM: Dulbecco’s modified Eagle’s medium
DPP4: Dipepidyl peptidase 4
DPP4: Dipepidyl peptidase 4
ELISA: Enzyme-linked immune sorbent assay
FBG: Fasting blood glucose
FBS: Fetal bovine serum
FINS: Fasting serum insulin
HFD: High-fat diet
HOMA-IR: Homeostasis model of assessment for IR index
IPITT: Intraperitoneal insulin tolerance test
IR: Insulin resistance
miR-214: MicroRNA-214
MiRNAs: MicroRNAs
miR-NC: MiRNA negative control
NC: Normal control
OGTT: Oral glucose tolerance test
PA: Palmitic acid
qRT-PCR: Quantitative real-time-PCR
ROC: Receiver operating characteristic curve
SD: Sprague-Dawley
VG: Vildagliptin
